# Natural Increases in Parasitoid and Predator Abundances and a Shift in Species Dominance Point to Improved Suppression of the Sorghum Aphid Since Its Invasion into North America

**DOI:** 10.3390/insects15120958

**Published:** 2024-12-02

**Authors:** Pius A. Bradicich, Ashleigh M. Faris, John W. Gordy, Michael J. Brewer

**Affiliations:** 1 Department of Entomology, Texas A&M AgriLife Research, 10345 State HWY 44, Corpus Christi, TX 78406, USA; pius.bradicich@ag.tamu.edu; 2 Department of Entomology & Plant Pathology, Oklahoma State University, 127 Noble Research Center, Stillwater, OK 74078, USA; ashleigh.faris@okstate.edu; 3 Syngenta Crop Protection, 410 Swing Rd., Greensboro, NC 27409, USA; john.gordy.ag@gmail.com

**Keywords:** Aphididae, Aphelinidae, Coccinellidae, *Melanaphis sorghi*, natural enemies, parasitoids, predators

## Abstract

*Melanaphis sorghi* (Theobald) (Hemiptera: Aphididae), commonly called the sorghum aphid, is an invasive pest of sorghum (*Sorghum bicolor*) (L.) in North America. It was first observed in 2013 along the Gulf Coastal Plains ecoregion of Texas, Louisiana (USA), and Mexico, where it quickly established itself as an economically important pest within a few years. This ecoregion contains an established complex of aphid natural enemies, including both predators and parasitoids. In the decade since its invasion, indicators of increased suppression observed across six years and five locations from south to north Texas were as follows: (1) aphid abundances trending downwards across the years, (2) overall natural enemy abundances trending upwards during the same time period, and (3) a key parasitoid and coccinellid species increasing in dominance. Two key taxa, *Aphelinus nigritus* (Howard) (Hymenoptera: Aphelinidae) and six species of coccinellids (Coleoptera: Coccinellidae), were likely responsible for the majority of the pest’s suppression. In light of these findings, the importance of monitoring and stewarding natural enemies of invasive insect pests is discussed as part of a comprehensive strategy to measure and reduce the impact of a pest invasion in large-scale agroecosystems.

## Simple Summary

The sorghum aphid is an invasive pest of grain sorghum grown in North America that was first observed in 2013 along the Gulf Coastal Plains ecoregion of Texas, Louisiana (USA), and Mexico. In the decade since its invasion, results point to increasing suppression of the sorghum aphid as most likely attributable to a native complex of predators and parasitoids that have shifted spatially and temporally. Indicators of increased suppression observed across six years and five locations from south to north Texas were as follows: (1) aphid abundances trending downwards across the years, (2) overall natural enemy abundances trending upwards during the same time period, and (3) a key parasitoid and coccinellid species increasing in dominance. In light of these findings, the importance of monitoring and stewarding natural enemies of invasive insect pests is discussed as part of a comprehensive strategy to measure and reduce the impact of a pest invasion in large-scale agroecosystems.

## 1. Introduction

The sorghum aphid, *Melanaphis sorghi* (Theobald) (Hemiptera: Aphididae), is an invasive pest of sorghum, *Sorghum bicolor* (L.), in North America. Thought to have originated in Africa or Asia, *M. sorghi* was first observed in 2013 along the Gulf Coastal Plains ecoregion of Texas, Louisiana (USA), and Mexico [1]. Since its initial appearance, the species quickly expanded its range to include 17 states in the USA and the eastern Gulf region of Mexico and the Caribbean [2]. Previously misidentified as the sugarcane aphid, *Melanaphis sacchari* (Zehntner), recent research has reclassified the species as a distinct superclone with feeding preferences for sorghum and the closely related Johnson grass (*Sorghum halepense* (L.)) instead of sugarcane (*Saccharum officinarum* (L.)) [3,4]. *M. sorghi* causes both direct and indirect damage to sorghum, potentially resulting in a modest yield decline to total crop loss [5]. Direct loss is caused by the aphid feeding on sorghum leaves and using their stylets to penetrate the plant tissues and remove phloem for nutrition, thereby weakening the plant and reducing the number and quality of seeds produced. Indirect economic loss is caused by the honeydew excreted by the aphids, which is a medium for sooty mold growth on leaves that inhibits photosynthesis. Honeydew, when abundant, can clog harvest equipment, delaying harvest [1,6]. To counter the crop losses caused by *M. sorghi* during the early disruptive phase of the invasion, protocols were developed to control the pest through chemical and cultural control practices [1,7,8].

Concurrently, some regions monitored natural enemies that began to feed on *M. sorghi* within a few years of establishment. These natural enemies consisted of predators and parasitoids, including coccinellids, chrysopids, hemerobiids, and syrphids, as well as aphelinid and braconid wasps [9,10,11]. In Texas, the most abundant primary parasitoid was *Aphelinus nigritus* (Howard) (Hymenoptera: Aphelinidae), although another primary parasitoid, *Lysiphlebus testaceipes* (Cresson) (Hymenoptera: Braconidae), and a hyperparasitoid were sometimes detected in high numbers [11]. The two primary parasitoid species produce the recognizable black (*A. nigritus*) and brown (*L. testaceipes*) aphid mummies that have been used for the assessment of sorghum aphid parasitism in the field. Predators have also been detected preying on *M. sorghi*, particularly coccinellids and syrphids [9,11].

Pest suppression by native and long-established resident natural enemies is an ideal economic and ecological solution for insect pest invasions because the biological control they provide is accomplished at costs reduced to monitoring activities and with reduced need for insecticides [12]. For aphid management in cereal crops, natural enemies have often been compatible with host plant resistance, including sorghum resistance to *M. sorghi* [13,14]. Broadly, natural enemies are estimated to provide close to half of all pest control in croplands, and the ecosystem services they provide are valued in the tens of millions of dollars each year within the USA [15,16,17]. Both predators and parasitoids modulate pest suppression. Their coexistence can lead to either positive or negative impacts on their ability to regulate pest populations [17]. For instance, predators and parasitoids can prey on pest insects at different life stages, resulting in a synergistic effect that enhances the suppression of a target pest [17,18]. In contrast, intraguild predation between natural enemies can cause suppression to decrease, such as when immature parasitoids within aphid mummies are consumed by predators, thereby reducing the impact of the parasitoid on aphid populations [17,19]. Overall, predators and parasitoids working together may suppress pests by themselves or in conjunction with other aphid management approaches. For invasive pests, the time sequence of adaptation of natural enemies to the new pest prey item is crucial in determining the economic impact of the pest invasion and the need for additional management inputs to control the pest invasion.

Evidence for *M. sorghi* suppression by natural enemies exists using several forms of experimental manipulation of predators, parasitoids, and *M. sorghi*. Some natural enemy taxa contributed more to suppression than others [9,14,20]. However, how suppression has changed temporally and spatially and whether natural enemy taxa respond differently are particularly relevant to pest invasions in field crops that are widely planted in large-scale agroecosystems. In this study, data on natural enemy and aphid populations collected from five different locations in Texas across a period of six years were compiled and analyzed. The objectives of these analyses were to evaluate (1) if the frequencies of natural enemies in six key taxa were equally represented in the five locations, (2) if the abundances of *M. sorghi* and two dominant natural enemy taxa (*A. nigritus* and coccinellids) differed across years and locations, and (3) if changes in natural enemy abundances between those two dominant taxa were reflected in the ratios of natural enemy to aphid abundances for each location-year. Based on the findings, the importance of monitoring and stewarding natural enemies of invasive insect pests is discussed as part of a comprehensive strategy to measure and reduce the impact of a pest invasion in large-scale agroecosystems.

## 2. Materials and Methods

### 2.1. Data Collection

**Figure 1:**
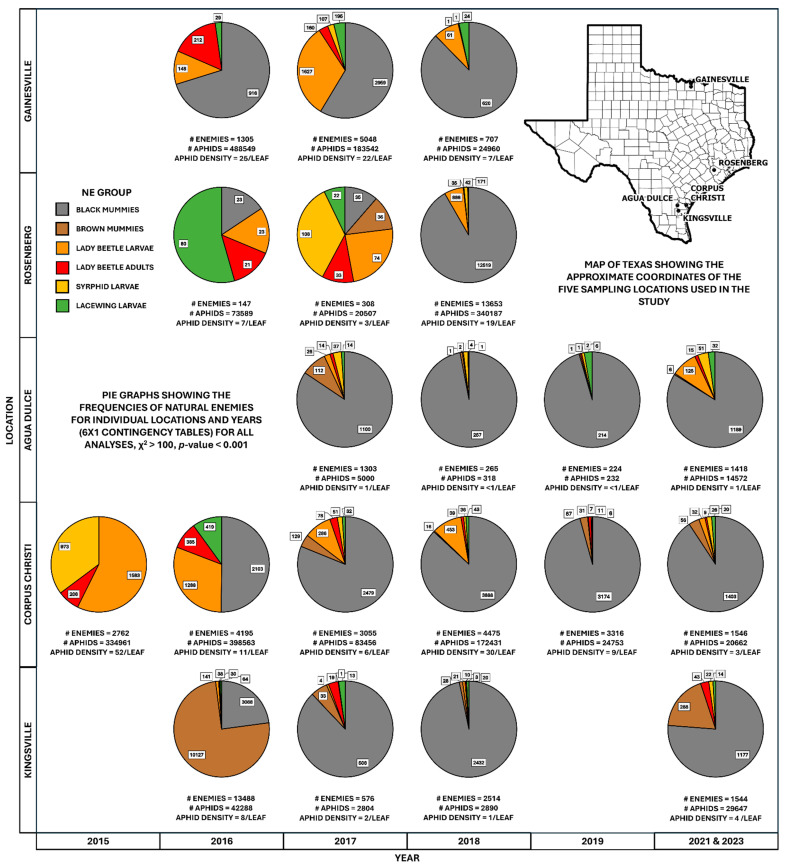
Pie graphs depicting the change in natural enemy frequencies for each location and year combination. The recovered number of taxa (# at bottom) and specific natural enemy taxa (boxed numbers) are given for each chart. Each pie slice depicts the frequency of each natural enemy (NE) group from the whole. For all individual 6 (group) by 1 (location year) contingency tables, χ^2^ > 100, and *p*-values < 0.001. A total of 20 location years were analyzed. Empty spaces indicate location years that were not sampled. The inset map of Texas depicts the approximate coordinates of the five sampling locations used in the study.

A data set from 2015 to 2023 was compiled from insect monitoring activities in five areas in Texas, USA. The locations, ranging from the most northern to the southernmost, were Gainesville, Rosenberg, Agua Dulce, Corpus Christi, and Kingsville, although each location was not represented each year (Figure 1). Data collection began when *M. sorghi* was first detected in sorghum fields (between April and May, depending on location) and continued until the aphid was no longer detected or the fields were harvested (between July and August). Data were collected on a weekly basis by examining a single upper and lower leaf of randomly selected sorghum plants. The number of plants examined at a sampling location on any given day ranged from 20 to 40. The data consisted of insect counts, which were recorded visually and focused on *M. sorghi* and key natural enemy taxa. These key taxa were black (*A. nigritus*) and brown (*L. testaceipes*) mummies (Hymenoptera: Aphelinidae; Braconidae), six species of lady beetle larvae and adults (Coleoptera: Coccinellidae), syrphid larvae (Diptera: Syrphidae), and lacewing larvae (Neuroptera: Chrysopidae). The six species of coccinellids were *Coccinella septempunctata*, *Coleomegilla maculata*, *Harmonia axyridis*, *Hippodamia convergens*, *Cycloneda sanguinea*, and *Olla v-nigrum*. Parasitoids were field-identified by the presence of black and brown aphid mummies and predators by the presence of adult (coccinellids only) and larval (all taxa) life stages [11]. Photographs of *M. sorghi* and its natural enemies are available [11,21].

### 2.2. Statistical Methods

Prior to analysis, the data were normalized to the smallest common leaf observation for each location and year (1068 leaves) because of differences in sampling frequency. Natural enemy data were simplified to six key groups that were relevant to *M. sorghi* ecology [11]. These were black (group 1) and brown (group 2) mummies, lady beetle (coccinellid) adults (group 3) and larvae (group 4), syrphid larvae (group 5), and lacewing larvae (group 6). To test if these key groups were represented equally in each of the 20 location-year combinations, 6 (group) by 1 (specific location and year) contingency tables were built (2021 and 2023 data were combined due to smaller sample sizes occurring in those years). Additionally, three 6 (year) by 5 (location) contingency tables focused on *M. sorghi* and the two most dominant natural enemy taxa (*A. nigritus*, adult and larval coccinellids) to test if each taxa’s abundance exhibited differences across locations and years. Finally, three more 6 (year) by 5 (location) contingency tables were built using the ratios of natural enemies to aphids to test if the natural enemy abundance adjusted for *M. sorghi* abundance shifted across locations and years. All analyses were conducted using JMP (2024) statistical software.

## 3. Results

### 3.1. 6 × 1 Contingency Tables—Key Natural Enemy Groups

The results from each of the 20, 6 (group) by 1 (specific location–year) contingency tables were significant (all χ^2^ values were greater than 100 and *p*-values were less than 0.001), indicating that the frequencies of the six natural enemy groups under consideration were not represented equally for each location–year combination. These results indicated a high likelihood that some natural enemy taxa were dominant over others and that taxa composition shifted across locations and years (Figure 1). There was an indication of a latitudinal gradient, with natural enemy taxa appearing relatively more diverse in the more northern sampling locations (Gainesville and Rosenberg) when compared with more southern locations where a few taxa tended to be detected more frequently, especially during the 2015–2017 sampling years (Figure 1). During that same period, the composition of natural enemy taxa shifted from a diverse assortment to a few key taxa. Overall, the parasitoid *A. nigritus* and, to a lesser degree, the coccinellid predators dominated the natural enemy complex numerically.

### 3.2. 6 × 5 Contingency Tables—Sorghum Aphids and Dominant Natural Enemies

The 6 (year) by 5 (location) contingency tables built for *M. sorghi* and both of its dominant natural enemy taxa, the coccinellids and *A. nigritus*, were significant (for all analyses, χ^2^ > 1000, *p*-value < 0.001). These results indicate that the abundances of both *M. sorghi* and its dominant natural enemies shifted across locations and years. For *M. sorghi*, larger abundances on average were observed in earlier years, likely due to the natural enemy populations lagging behind those of the aphid during its initial invasion phase. Similarly, the coccinellids also experienced larger abundances on average in earlier years. Finally, the abundances of *A. nigritus* were likewise affected by location and year. However, they did not fluctuate as much as the other taxa, particularly after 2016 (Figure 1).

### 3.3. 6 × 5 Contingency Tables—Dominant Natural Enemy and Aphid Ratios

The results for the two 6 (year) by 5 (location) contingency tables built to evaluate if the ratio of dominant natural enemies to *M. sorghi* differed between location–years were both insignificant (χ^2^ = 0.61, *p*-value = 1.00, and χ^2^ = 0.07, *p*-value = 1.00, for *A. nigritus* and coccinellids, respectively). These results indicate that the ratio of *A. nigritus* and the coccinellids rose in years when the *M. sorghi* population declined and that there was an overall trend of increasing natural enemy to aphid ratios across the years. The insignificance of these results supports that the ratios of both natural enemy taxa to *M. sorghi* were resistant to change and remained relatively consistent across years and locations despite the insect’s populations experiencing fluctuations in size during the same period.

## 4. Discussion

Prior studies have revealed that the natural enemy complex present in sorghum is similar across a large swathe of the USA, ranging from south Texas to southern Kansas [9,11,14]. What is not known is how this community has changed over the years since *M. sorghi* first arrived and if this change is related to the increased suppression of that pest. The highly significant results from each of the twenty contingency tables constructed to analyze the six key natural enemy groups in the first analysis (Section 3.1) strongly indicated that the frequencies of these taxa were not equally represented over the years examined for each location, although they did not provide any information on the contribution to the suppression of individual taxa. Over time, the natural enemy frequencies changed from a more diverse community to the dominance of one parasitoid, *A. nigritus*, and, to a lesser extent, coccinellids in the more northern sites within a few years (Figure 1). This result is not unexpected as *A. nigritus* has been shown to be the most effective agent of biological control against *M. sorghi*, and it is expected that its abundance within the natural enemy complex would increase as it adapted to a new prey source [13,14]. This result resembles that of a study with another cereal aphid, *Diuraphis noxia*, where the parasitoid *Aphelinus albipodus* became the dominant natural enemy through a multi-year study [22]. However, the contributions of predators to aphid suppression should not be trivialized, as studies have shown that suppression is enhanced when predators and parasitoids work together [18,23,24]. Finally, the compatibility of *A. nigritus* and coccinellids may be particularly relevant in the more northern locations of this study, as seen in cage exclusion studies of *M. sorghi* and its natural enemies conducted in Oklahoma and south Texas [14].

**Figure 2:**
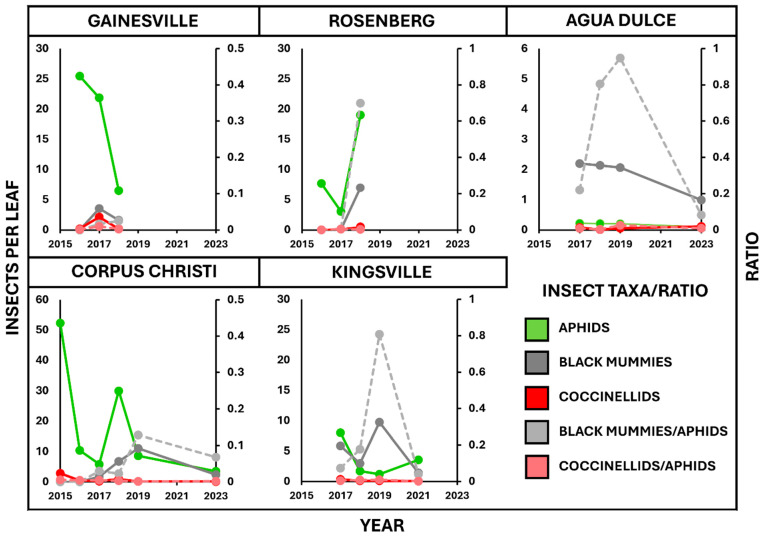
Line graphs showing *M. sorghi* and dominant natural enemy population changes across the years for each of the five locations: Solid lines show insects (*M. sorghi*, *A. nigritus,* and coccinellids) per leaf, and the dashed lines show the ratio of dominant natural enemies (*A. nigritus* and coccinellids) to *M. sorghi*. For interpretability, natural enemy data have been inflated by a factor of ten.

The results of the second analysis (Section 3.2) revealed that all three insect taxa investigated, *M. sorghi*, *A. nigritus*, and the six species of coccinellids, showed significant year-to-year and location-to-location shifts in abundance. Broadly, *A. nigritus* was more abundant in the more southern locations (Agua Dulce, Corpus Christi, and Kingsville), while the more northern and central locations (Gainesville and Rosenberg) had relatively more coccinellids present (Figure 1). This is understandable, as studies have shown that the influence of predators on *M. sorghi* suppression is greater in more northern locations such as Oklahoma [24,25]. Larger populations of coccinellids in earlier years may be explained by the ability of generalist predators to adapt more quickly to new resources than specialists [9]. In general, the *A. nigritus* and coccinellid populations would rise and fall in synchrony with the *M. sorghi* population (Figure 2), approximating the classic arthropod predator-prey model [26]. This result is similar to the predator-prey dynamics observed in agricultural and natural systems [12]. Interestingly, the results also revealed that the abundances of the dominant parasitoid, *A. nigritus*, did not appear to fluctuate as much between location years as the other taxa and were the numerically dominant taxa from 2017 onwards (Figure 1). The likely explanation for this observation is that once *A. nigritus* had adapted to utilizing *M. sorghi* as a prey source, it was able to exploit the aphid as prey once the pest first infested annually planted sorghum fields each year. *A. nigritus* has been observed when sorghum is not in cultivation in both crop fields and non-crop areas containing ratoon sorghum and Johnson grass [27]. A relatively stable presence of the parasitoid in croplands and semi-natural areas may allow a quick response to reinvasion by *M. sorghi* of new sorghum crops each year.

The third analysis (Section 3.3) examining the ratios of two dominant natural enemy taxa (*A. nigritus* and the coccinellids) was not significant, indicating that the ratios of these natural enemy groups were relatively stable across locations and years. This result likely reflected that once these natural enemies had adapted to *M. sorghi* as a prey source, they improved in their synchrony and responsiveness to *M. sorghi.* The proportional natural enemy to aphid ratio remained relatively constant across locations and years, even though the actual abundance of the insects may have fluctuated considerably during the same time period. Broadly, *M. sorghi* populations experienced a downward trend in abundance in the same locations and years that the *A. nigritus* and coccinellid populations experienced an upward trend. This can be attributed to the populations of these natural enemies responding to growing populations of their prey by maximizing their reproductive output [28]. These trends can be visualized in Figure 2, especially at the Corpus Christi, Rosenberg, and Gainesville locations.

Based on the results of this study, the implementation of stewardship practices such as judicious selective insecticide use, habitat conservation, and fostering biodiversity may help ensure that natural enemies are able to thrive and maximize *M. sorghi* suppression. Some studies have shown that aphid control can be increased when insecticides are used alongside specific natural enemy groups [29]. However, it is best to only employ insecticides when necessary to prevent harming beneficial species [30,31]. Conservation biological control can be used for *M. sorghi* by maintaining habitat for its natural enemies as part of standard practice around sorghum fields. Within the sorghum agroecosystem of Texas, this would be best accomplished by maintaining hedgerows or grassy strips containing flowering and perennial plants that provide shelter and overwintering sites for natural enemies around the perimeters of crop fields [32,33]. These conservation activities may enhance biodiversity in general, which may support healthy populations of beneficial insects that may quickly respond to aphid infestations, including *M. sorghi* on sorghum. For example, *Hippodamia* spp. (Coleoptera: Coccinellidae) have been observed to feed on the early-emerging corn leaf aphids (*Rhopalosiphum maidis*) and build their populations before switching to the economically important greenbug (*Scizaphis graminum*) [34]. A similar phenomenon likely occurs in Texas with both parasitoids and predators feeding on alternative species before the emergence of *M. sorghi* into sorghum fields. Parasitoids of *M. sorghi* have been found in non-crop habitats around sorghum before planting and continuing after harvest [27]. This is an indication that extant practices in this system may be contributing to the conservation and biological control of *M. sorghi*. Although using augmentative biological control to suppress cereal aphid populations has been proposed in the past in North America [35], the additional input for *M. sorghi* is likely unnecessary, as both *A. nigritus* and *L. testaceipes* are able to successfully overwinter in the region and are found in non-crop habitat surrounding sorghum fields [27,35,36].

## 5. Conclusions

The results of this study highlight the shifts in abundance and species composition experienced by the natural enemy complex preying on *M. sorghi* across a decade since its invasion of North America. The results provide evidence on how natural enemies already present within the ecosystem can be pre-adapted to prey on emerging pest species and quickly respond to yearly reinvasions of the pest on annually planted crops. For *M. sorghi* populations within the widespread sorghum agroecosystem of Texas, different natural enemy taxa contributed to suppression, with certain taxa being more prevalent in some locations (for example, a greater presence of coccinellids in more northern locations). The results also highlight the importance of monitoring natural enemies in large-scale agroecosystems. This is because through tracking the populations of predators and parasitoids, growers can evaluate if and when the regulation of *M. sorghi* by natural enemies needs to be supplemented in the near term (through insecticide use) or the long term in areas of consistently low natural enemy activity (by planting sorghum hybrids that are partially resistant to *M. sorghi*) [13,37,38]. Natural enemies of *M. sorghi* have become a key component of a comprehensive strategy to reduce the impact of the invasion by *M. sorghi* in the large-scale sorghum agroecosystems of North America.

## Data Availability

The data presented in this study are available on request from the corresponding author.

## References

[1] Bowling R.D., Brewer M.J., Kerns D.L., Gordy J., Seiter N., Elliott N.E., Buntin G.D., Way M., Royer T., Biles S. (2016). Sugarcane aphid (Hemiptera: Aphididae): A new pest on sorghum in North America. J. Integr. Pest Manag..

[2] Harris-Shultz K., Ni X., Wadl P.A., Wang X., Wang H., Huang F., Flanders K., Seiter N., Kerns D., Meagher R. (2017). Microsatellite markers reveal a predominant sugarcane aphid (Homoptera: Aphididae) clone is found on sorghum in seven states and one territory of the USA. Crop Sci..

[3] Nibouche S., Costet L., Medina R.F., Holt J.R., Sadeyen J., Zoogones A.-S., Brown P., Blackman R.L. (2021). Morphometric and molecular discrimination of the sugarcane aphid, *Melanaphis sacchari* (Zehntner, 1897) and the sorghum aphid *Melanaphis sorghi* (Theobald, 1904). PLoS ONE.

[4] Esquivel I.L., Faris A.M., Brewer M.J. (2021). Sugarcane aphid, *Melanaphis sacchari* (Hemiptera: Aphididae), abundance on sorghum and johnsongrass in a laboratory and field setting. Crop Prot..

[5] Zapata S.D., Dudensing R., Sekula D., Esparza-Díaz G., Villanueva R. (2018). Economic impact of the sugarcane aphid outbreak in South Texas. J. Agric. Appl. Econ..

[6] Vasquez A., Belsky J., Khanal N., Puri H., Balakrishnan D., Joshi N.K., Louis J., Studebaker G., Kariyat R. (2024). *Melanaphis sacchari/sorghi* complex: Current status, challenges and integrated strategies for managing the invasive sap-feeding insect pest of sorghum. Pest Manag. Sci..

[7] Gordy J.W., Brewer M.J., Bowling R.D., Buntin G.D., Seiter N.J., Kerns D.L., Reay-Jones F.P., Way M. (2019). Development of economic thresholds for sugarcane aphid (Hemiptera: Aphididae) in susceptible grain sorghum hybrids. J. Econ. Entomol..

[8] Gordy J.W., Seiter N.J., Kerns D.L., Reay-Jones F.P., Bowling R.D., Way M., Brewer M.J. (2021). Field assessment of aphid doubling time and yield of sorghum susceptible and partially resistant to sugarcane aphid (Hemiptera: Aphididae). J. Econ. Entomol..

[9] Colares F., Michaud J., Bain C.L., Torres J.B. (2015). Recruitment of aphidophagous arthropods to sorghum plants infested with *Melanaphis sacchari* and *Schizaphis graminum* (Hemiptera: Aphididae). Biol. Control.

[10] Brewer M.J., Gordy J.W., Kerns D.L., Woolley J.B., Rooney W.L., Bowling R.D. (2017). Sugarcane aphid population growth, plant injury, and natural enemies on selected grain sorghum hybrids in Texas and Louisiana. J. Econ. Entomol..

[11] Maxson E.L., Brewer M.J., Rooney W.L., Woolley J.B. (2019). Species composition and abundance of the natural enemies of sugarcane aphid, *Melanaphis sacchari* (Zehnter) (Hemiptera: Aphididae), on sorghum in Texas. Proc. Entomol. Soc. Wash..

[12] Power A.G. (2010). Ecosystem services and agriculture: Tradeoffs and synergies. Philos. Trans. R. Soc. B Biol. Sci..

[13] Brewer M.J., Peairs F.B., Elliott N.C. (2019). Invasive cereal aphids of North America: Ecology and pest management. Annu. Rev. Entomol..

[14] Faris A.M., Elliott N.C., Brewer M.J. (2022). Suppression of the sugarcane aphid, *Melanaphis sacchari* (Hemiptera: Aphididae), by resident natural enemies on susceptible and resistant sorghum hybrids. Environ. Entomol..

[15] Pimentel D. (2005). Environmental and economic costs of the application of pesticides primarily in the United States. Environ. Dev. Sustain..

[16] Losey J.E., Vaughan M. (2006). The economic value of ecological services provided by insects. Bioscience.

[17] Dainese M., Schneider G., Krauss J., Steffan-Dewenter I. (2017). Complementarity among natural enemies enhances pest suppression. Sci. Rep..

[18] Snyder W.E., Ives A.R. (2003). Interactions between specialist and generalist natural enemies: Parasitoids, predators, and pea aphid biocontrol. Ecology.

[19] Colfer R., Rosenheim J. (2001). Predation on immature parasitoids and its impact on aphid suppression. Oecologia.

[20] Alhadidi S.N., Griffin J.N., Fowler M.S. (2018). Natural enemy composition rather than richness determines pest suppression. BioControl.

[21] Faris A.M., Brewer M.J. Natural Enemies of the Sugarcane Aphid on Sorghum in South Texas. https://agrilifelearn.tamu.edu/s/product/natural-enemies-of-the-sugarcane-aphid-on-sorghum-in-south-texas/01t4x000004OUcTAAW.

[22] Brewer M.J., Noma T., Elliott N.C. (2005). Hymenopteran parasitoids and dipteran predators of the invasive aphid *Diuraphis noxia* after enemy introductions: Temporal variation and implication for future aphid invasions. Biol. Control.

[23] Snyder W.E., Ballard S.N., Yang S., Clevenger G.M., Miller T.D., Ahn J.J., Hatten T.D., Berryman A.A. (2004). Complementary biocontrol of aphids by the ladybird beetle *Harmonia axyridis* and the parasitoid *Aphelinus asychis* on greenhouse roses. Biol. Control.

[24] Elliott N., Giles K., Brewer M., Szczepaniec A., Knutson A., Michaud J., Jessie C., Faris A., Elkins B., Wang H.-H. (2021). Recruitment of natural enemies of the invasive sugarcane aphid vary spatially and temporally in sorghum fields in the Southern Great Plains of the USA. Southwest. Entomol..

[25] Faris A.M., Brewer M.J., Elliott N.C., Brewer M.J., Hein G.L. (2024). Natural Enemy Suppression Supplemented by Regional Pest Management for the Invasive *Melanaphis sorghi*, Sorghum Aphid, on Sorghum. Arthropod Management and Landscape Considerations in Large-Scale Agroecosystems.

[26] Hassell M.P. (1978). The Dynamics of Arthropod Predator-Prey Systems.

[27] Faris A.M., Brewer M.J., Elliott N.C. (2022). Parasitoids and predators of the invasive aphid *Melanaphis sorghi* found in sorghum and non-crop vegetation of the sorghum agroecosystem. Insects.

[28] Kindlmann P., Dixon A.F., Kindlman P., Dixon A.F.G., Michaud J.P. (2010). Modelling Population Dynamics of Aphids and their Natural Enemies. Aphid Biodiversity Under Environmental Change: Patterns and Processes.

[29] Hakeem A., Parajulee M. (2019). Integrated management of sugarcane aphid, *Melanaphis sacchari* (Hemiptera: Aphididae), on sorghum on the Texas high plains. Southwest. Entomol..

[30] Ruberson J., Nemoto H., Hirose Y. (1998). Pesticides and Conservation of Natural Enemies in Pest Management. Conservation Biological Control.

[31] Okosun O.O., Allen K.C., Glover J.P., Reddy G.V. (2021). Biology, ecology, and management of key sorghum insect pests. J. Integr. Pest Manag..

[32] Nentwig W., Frank T., Lethmayer C. (1998). Sown Weed Strips: Artificial Ecological Compensation Areas as an Important Tool in Conservation Biological Control. Conservation Biological Control.

[33] Ferro D.N., McNeil J.N. (1998). Habitat Enhancement and Conservation of Natural Enemies of Insects. Conservation Biological Control.

[34] Royer T.A., Pendleton B.B., Elliott N.C., Giles K.L. (2015). Greenbug (Hemiptera: Aphididae) biology, ecology, and management in wheat and sorghum. J. Integr. Pest Manag..

[35] Fernandes O.A., Wright R.J., Mayo Z. (1998). Parasitism of greenbugs (Homoptera: Aphididae) by *Lysiphlebus testaceipes* (Hymenoptera: Braconidae) in grain sorghum: Implications for augmentative biological control. J. Econ. Entomol..

[36] Giles K., Elliott N.C., Royer T., Butler H., Rudin N., Brewer M.J., Hein G.L. (2024). Ecology of Aphid Parasitoids in Winter Wheat Habitats of the Southern Plains: How Latitude and Crop Diversity Influence Pest Management. Arthropod Management and Landscape Considerations in Large-Scale Agroecosystems.

[37] Thudi M., Reddy M.S., Naik Y.D., Cheruku V.K.R., Sangireddy M.K.R., Cuevas H.E., Knoll J.E., Louis J., Kousik C.S., Toews M.D. (2024). Invasive sorghum aphid: A decade of research on deciphering plant resistance mechanisms and novel approaches in breeding for sorghum resistance to aphids. Crop Sci..

[38] Giles K.L., Jones D.B., Royer T.A., Elliott N.C., Kindler S.D. (2003). Development of a sampling plan in winter wheat that estimates cereal aphid parasitism levels and predicts population suppression. J. Econ. Entomol..

